# VExUS to Guide Ultrafiltration in Hemodialysis: Exploring a Novel Dimension of Point of Care Ultrasound

**DOI:** 10.24908/pocus.v9i1.16985

**Published:** 2024-04-22

**Authors:** Abhilash Koratala, Mohamed Ibrahim, Sirisha Gudlawar

**Affiliations:** 1 Division of Nephrology, Medical College of Wisconsin Milwaukee, WI USA

**Keywords:** POCUS, Dialysis, Congestion, VExUS

## Abstract

Venous Excess Ultrasound (VExUS) is a valuable bedside tool for nephrologists within a multi-organ point of care ultrasound (POCUS) framework. VExUS can address limitations of conventional physical examination in identifying hemodynamic congestion and monitoring treatment efficacy. A 53-year-old man with heart failure and end-stage kidney disease on hemodialysis presented with elevated liver function tests. Despite an unremarkable right upper quadrant ultrasound done by radiology, the review of images by the nephrology team uncovered severe venous congestion, evidenced by a dilated inferior vena cava (IVC) and abnormal hepatic and portal vein flow. Follow-up assessments included VExUS scans and daily ultrafiltration that resulted in a notable 8-liter fluid removal. The dynamic changes in IVC shape and improvement in Doppler waveforms underscored successful decongestion. This case demonstrates the clinical utility of VExUS in guiding therapy for fluid overload in complex patients.

## Introduction

**Figure 1:**
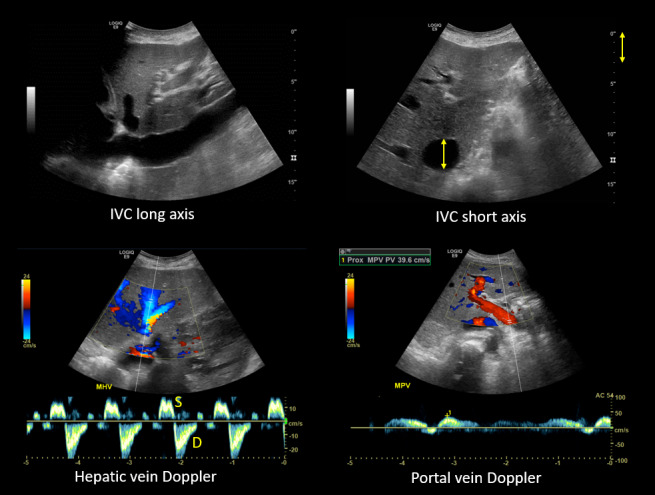
1: Radiology-performed scan images demonstrating a dilated inferior vena cava (approximately 3 cm), S-wave reversal on hepatic vein Doppler and a pulsatile portal vein (below-the-baseline blebs represent flow reversal).

Venous Excess Ultrasound (VExUS) is a novel application of point of care ultrasound (POCUS). It allows for the assessment of systemic venous congestion, which is a function of elevated right atrial pressure and reduced venous compliance 1. VExUS involves evaluating alterations in Doppler waveforms of hepatic, portal, and intrarenal veins to quantify congestion into three grades, as summarized in Figure 1. Due to the dynamic nature of these waveforms, VExUS is valuable for both diagnosing venous congestion and monitoring the effectiveness of decongestive therapy 2. Traditional physical examination measures for detecting congestion present various constraints, especially in individuals with heart failure and those undergoing hemodialysis 3, 4. VExUS, integrated into a multi-organ POCUS strategy, offers an additional tool at the bedside for nephrologists 5. This case shows where VExUS helped ensure adequate decongestion in a patient on hemodialysis. 

## Case Report

A 53 year old man with heart failure with reduced ejection fraction (~37%) secondary to non-ischemic cardiomyopathy and end-stage kidney disease (ESKD) recently initiated on hemodialysis underwent a right upper quadrant ultrasound for elevated liver function tests. Despite a radiology report indicating "normal liver morphology and hemodynamics," a review of images by the nephrology team revealed severe venous congestion. This was evidenced by a dilated inferior vena cava (IVC) with an approximate anteroposterior diameter of 3 cm, systolic (S-wave) reversal in the hepatic vein flow, and a pulsatile portal vein with some flow reversal (Figure 1). 

**Figure 2:**
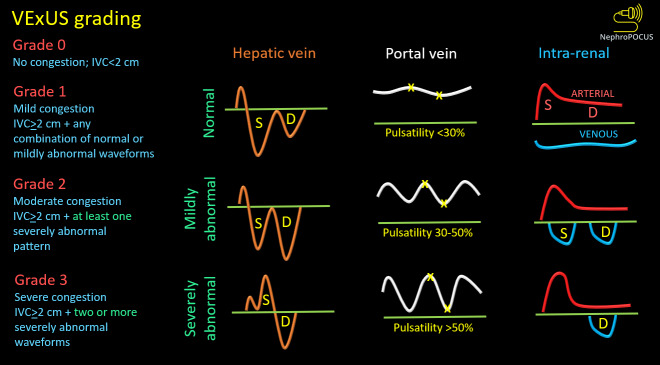
2: Venous excess ultrasound grading: When the diameter of the inferior vena cava is > 2 cm, three grades of congestion are defined based on the severity of abnormalities on hepatic, portal, and renal parenchymal venous Doppler. Hepatic vein Doppler is considered mildly abnormal when the systolic (S) wave is smaller than the diastolic (D) wave, but still below the baseline; it is considered severely abnormal when the S-wave is reversed. Portal vein Doppler is considered mildly abnormal when the pulsatility is 30% to 50%, and severely abnormal when it is ≥ 50%. Asterisks represent points of pulsatility measurement. Renal parenchymal vein Doppler is mildly abnormal when it is pulsatile with distinct S and D components, and severely abnormal when it is monophasic with D-only pattern. Adapted from NephroPOCUS.com with permission.

**Figure 3:**
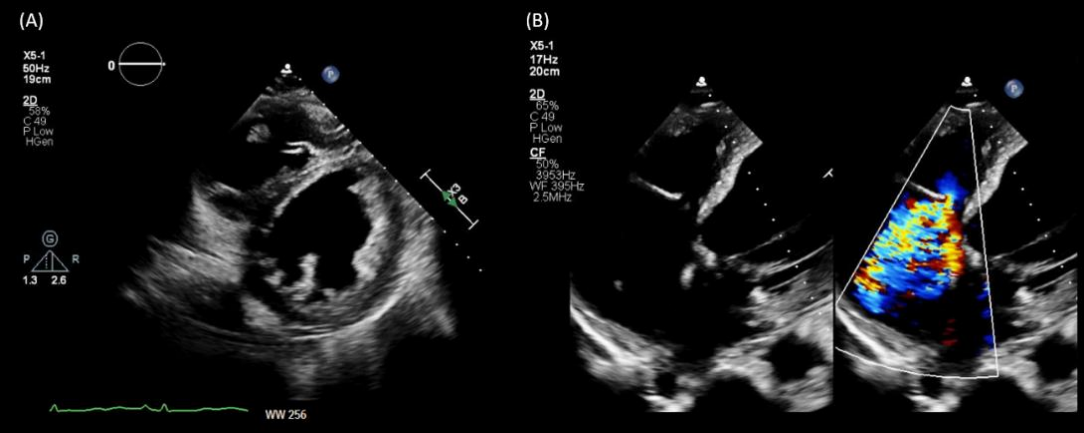
Formal echocardiogram images demonstrating (A) interventricular septal flattening on parasternal short axis view and (B) qualitatively severe tricuspid regurgitation.

**Figure 4:**
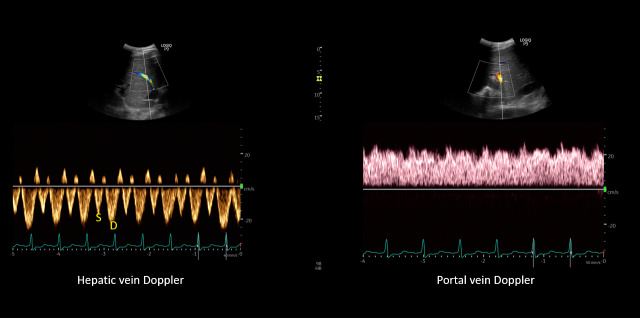
POCUS images demonstrating S<D pattern on hepatic vein Doppler and a normal appearing (pulsatility <30%) portal vein waveform.

**Figure 5:**
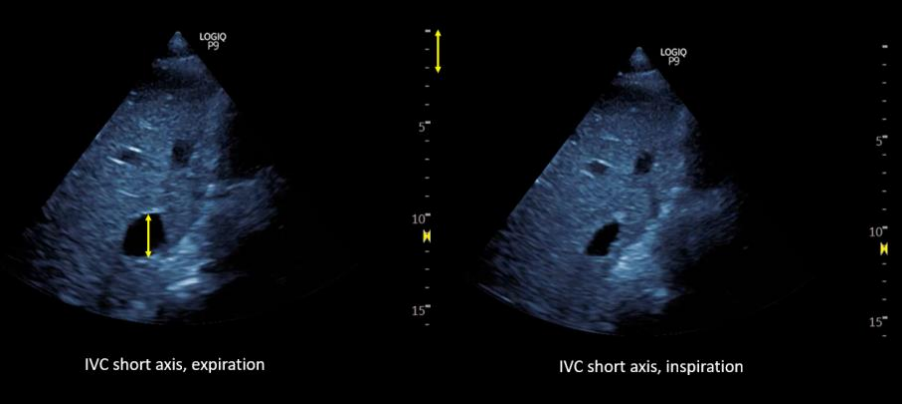
Inferior vena cava ultrasound short axis demonstrating the maximal anteroposterior diameter (approximately 2.1 cm) and inspiratory collapse.

**Figure 6:**
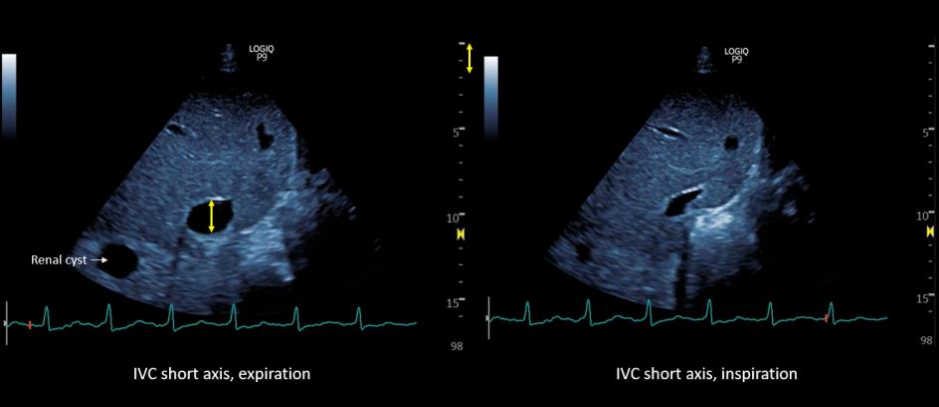
Inferior vena cava ultrasound short axis demonstrating the maximal anteroposterior diameter (approximately 1.9 cm) and inspiratory collapse.

**Figure 7:**
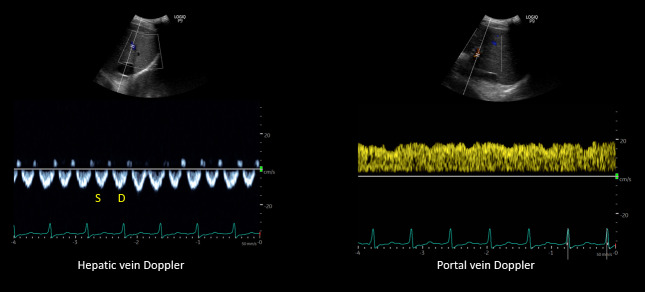
POCUS images demonstrating almost equal S and D waves on hepatic vein Doppler and a normal (pulsatility <30%) appearing portal vein waveform.

These sonographic findings are consistent with VExUS grade 3 (Figure 2). Interestingly, the patient lacked pedal edema or shortness of breath. A formal echocardiogram demonstrated reduction of left ventricular ejection fraction from a baseline of 37% to ~30%, new right ventricular enlargement with interventricular septal flattening (D-sign), and severe functional tricuspid regurgitation, suggestive of fluid overload (Figure 3). The patient history was not suggestive of pulmonary embolism. A nuclear medicine stress test was negative for ischemic changes. Over the subsequent three days, the nephrology team performed daily ultrafiltration, resulting in removal of 8 liters of fluid (net negative 4.5 liters on day 3). At the end of the 2nd session, the nephrology team performed a follow up VExUS scan that showed significant improvement in the congestion. The portal vein was completely normalized, whereas the hepatic vein showed mild congestion with S-wave less than D-wave. A simultaneous EKG tracing was used to avoid errors in misidentification of the waves (Figure 4). The IVC maximal diameter improved to approximately 2.1 cm, with >50% inspiratory collapse with an estimated right atrial pressure of 8 mmHg (Figure 5). Follow up POCUS after the 3rd session demonstrated further improvement in IVC size (<2 cm), and collapsibility consistent with an estimated right atrial pressure of 3 mmHg (Figure 6). Remarkably, the shape of the IVC shifted from circular to oval during the decongestion of the patient, which is a clinically useful qualitative parameter. Hepatic vein Doppler demonstrated further improvement in S-wave amplitude to near-normal configuration, and the portal vein remained continuous (Figure 7). Intrarenal venous Doppler was not performed, as it is unreliable in ESKD. 

Additionally, cardiac POCUS revealed a rounded left ventricle in the parasternal short-axis view. This indicated resolution of the D-sign along with significant improvement in tricuspid regurgitation (Video S1 and S2). Although serum transaminases showed improvement during this time (ALT and AST decreased from 184 to 98 U/L and 156 to 59 U/L, respectively), it cannot be solely attributed to reduction in congestion, as the patient was concurrently diagnosed with a hepatitis C infection (Hep C RNA 141,000 IU/mL). The patient's weight after the 3rd dialysis session was conveyed to his outpatient nephrologist to assist in adjusting dry weight. 

This case underscores key lessons: 1. Radiology reports may not encompass information on venous congestion, necessitating nephrologists' awareness of imaging findings related to systemic hemodynamics. 2. Patient symptoms and clinical signs might not correlate with hemodynamic congestion, and VExUS can serve as a valuable bed-side indicator in such instances (further research is warranted). 3. VExUS serves as a visual bedside guide for decongestion, enabling real-time interpretation and management by clinicians. 

## Disclosures

The authors declare no potential conflicts of interest. 

## Funding statement

Abhilash Koratala reports research funding from Kidney Cure and the American Society of Nephrology’s William and Sandra Bennett Clinical Scholars Grant. 

## Patient Consent

Informed consent has been obtained from the patient for the publication of this case study.

## Supplementary Material

Video S1 Cardiac POCUS parasternal short axis view demonstrating round left ventricle without interventricular septal flattening. Note that the systolic function of the left ventricle is reduced.

Video S2 Cardiac POCUS apical 4-chamber view showing minimal tricuspid regurgitation on color Doppler.
